# Dual-Filter Cross Attention and Onion Pooling Network for Enhanced Few-Shot Medical Image Segmentation

**DOI:** 10.3390/s25072176

**Published:** 2025-03-29

**Authors:** Lina Ni, Yang Liu, Zekun Zhang, Yongtao Li, Jinquan Zhang

**Affiliations:** 1 College of Computer Science and Engineering, Shandong University of Science and Technology, Qingdao 266590, China; nilina@sdust.edu.cn (L.N.); 202282060044@sdust.edu.cn (Y.L.); 202282060034@sdust.edu.cn (Y.L.); 2 Key Laboratory of the Ministry of Education for Embedded System and Service Computing, Tongji University, Shanghai 201804, China; 3 School of Computer Science, University of Glasgow, Glasgow G12 8QQ, UK; 2764399z@student.gla.ac.uk

**Keywords:** few-shot learning, medical image segmentation, prototype learning

## Abstract

Few-shot learning has demonstrated remarkable performance in medical image segmentation. However, existing few-shot medical image segmentation (FSMIS) models often struggle to fully utilize query image information, leading to prototype bias and limited generalization ability. To address these issues, we propose the dual-filter cross attention and onion pooling network (DCOP-Net) for FSMIS. DCOP-Net consists of a prototype learning stage and a segmentation stage. During the prototype learning stage, we introduce a dual-filter cross attention (DFCA) module to avoid entanglement between query background features and support foreground features, effectively integrating query foreground features into support prototypes. Additionally, we design an onion pooling (OP) module that combines eroding mask operations with masked average pooling to generate multiple prototypes, preserving contextual information and mitigating prototype bias. In the segmentation stage, we present a parallel threshold perception (PTP) module to generate robust thresholds for foreground and background differentiation and a query self-reference regularization (QSR) strategy to enhance model accuracy and consistency. Extensive experiments on three publicly available medical image datasets demonstrate that DCOP-Net outperforms state-of-the-art methods, exhibiting superior segmentation and generalization capabilities.

## 1. Introduction

Automatic medical image segmentation [1] has demonstrated significant advantages in terms of efficiency, flexibility, and reliability compared to manual segmentation with the evolution of artificial intelligence [2]. It provides a more scientific and accurate basis for physicians’ clinical decision making. Currently, most existing medical image segmentation models are built on deep learning methods [3,4,5]. An essential prerequisite for these models to achieve excellent performance is to have a large amount of annotated data during training. However, due to the necessity to protect patient privacy and the professionalism required to label medical images, constructing medical image datasets with dense annotations takes time and effort [6]. Therefore, medical image segmentation evolves into an immensely challenging endeavor.

Fortunately, few-shot learning (FSL) [7,8,9] offers a potential solution to address the issue of insufficient medical image data. The key to this method is to fully utilize the similarity between the support set and the query set to guide segmentation and effectively enhance the model’s “learn how to learn” capability, enabling it to quickly generalize to new categories even in the case of scarce samples. Since ALP-Net [10] introduces few-shot learning into medical image segmentation, few-shot medical image segmentation (FSMIS) models have developed rapidly. The current FSMIS models are mainly divided into two categories: one is based on prototype networks [11,12,13,14,15] and the other is based on interaction-based models [16,17,18,19,20]. Nowadays, in the FSMIS field, models based on prototype networks have become the mainstream architecture.

However, traditional prototype-based models [21,22] may fail to effectively learn from the query foreground (FG) features, which can amplify the prototype bias issue. To address this issue, the recent CRAP-Net [20] employs a multi-layer cycle-resemblance module to capture pixel-level relationships between support features and query features. Moreover, to eliminate irrelevant pixel information in support images, CAT-Net [23] adopts a cross masked attention mechanism to enable effective interaction between support FG features and query features. Regrettably, such approaches tend to intermingle the background (BG) features of the query image with the FG features of the support images, resulting in BG mismatches. Furthermore, some models rely solely on a masked average pooling (MAP) [24] method during prototype extraction after feature interactions. This process may cause prototypes to struggle to represent fine-grained support FG features. The combination of these two factors magnifies the prototype bias issue.

Meanwhile, existing FSMIS models [16,25,26] suffer from poor generalization performance and lack of in-depth learning from the query image. This limitation restricts their ability to effectively adapt to unseen categories. To address these issues, PA-Net [27] introduces a potential solution by incorporating a prototype alignment regularization task, which allows query image information to flow back to the support image, thereby enhancing the segmentation ability of prototype learning-based models. Nevertheless, its improvement in model generalization performance is limited. In addition, some existing methods [25,26] attempt to use the query image to generate learnable thresholds to delineate FG and BG regions when performing segmentation on the query image. However, these methods only take the query image as input, making the threshold vulnerable to noise. Meanwhile, due to the insufficient feature extraction of the query image, the generated thresholds are challenging to complete the delineation task effectively. Consequently, it is essential for elevating FSMIS models’ generalization and segmentation capabilities to design a regularization task and a practical threshold generation module.

To tackle the issues above, we propose a dual-filter cross attention and onion pooling network (DCOP-net) for FSMIS. Compared to other FSMIS methods that suffer from prototype bias and lack generalization ability, the innovation of DCOP-Net lies in the effective interaction between support and query features, allowing contextual information from support images to be better retained as prototypes, thereby addressing the BG mismatch problem and mitigating prototype bias. Furthermore, DCOP-Net is capable of generating robust thresholds for anomaly score discretization and incorporates a novel feedback mechanism, enabling superior segmentation and generalization performance compared to other FSMIS methods. Specifically, we design dual-filter cross attention (DFCA) and onion pooling (OP) modules in the prototype learning stage to alleviate the prototype bias problem. Firstly, the DFCA module performs a dual filter on the query BG by a prior mask and an adaptive value to avoid the problem of BG mismatch. Subsequently, the DFCA module reduces the intra-class gap of FG categories by feature fusion. It captures the pixel-wise relation between query FG features and support FG features using the cross attention mechanism to obtain enhanced support and query features. Inspired by real-life Russian nesting dolls, we construct the OP module which generates onion layer masks by eroding query masks layer by layer through the proposed erosion pooling operation. Then, the OP module utilizes MAP to extract the prototypes and a fully connected layer-based approach to enable the prototypes to learn from each other, thus reducing the loss of critical details of the medical images when obtaining the prototypes. In this way, the quality of the prototype is significantly improved.

In the segmentation stage, we propose a parallel threshold perception (PTP) module and implement query self-reference regularization (QSR) to improve the model’s generalization and segmentation capabilities. The PTP module learns segmentation thresholds based on a parallel architecture, which effectively improves the nonlinearity of the module and reduces the impact of noise in medical images on threshold generation. The QSR module improves the model’s self-correction and generalization ability by self-guiding the query prediction information flow back to the query image, forming a feedback mechanism between the prediction mask and itself.

In summary, our contributions can be summarized as follows:

1. In the prototype learning stage, we propose the DFCA and OP modules to mitigate the issue of prototype bias in FSMIS. The DFCA module employs a dual-filter mechanism to prevent BG mismatches, enabling prototypes to learn query FG information more efficiently. Meanwhile, the OP module preserves the prototype’s contextual information when extracting the contextual information of prototypes during extraction and enhances their ability to represent FG features in medical images.
2. In the segmentation stage, we develop the PTP and QSR modules to improve the accuracy and generalization capabilities of the FSMIS models when segmenting query images. PTP module acquires valid thresholds through a dual-path architecture. Meanwhile, QSR improves the model’s ability to adapt to unseen categories by forming a suitable feedback mechanism.
3. We propose an effective FSMIS model DCOP-Net. Extensive experiments on three publicly available medical image datasets show that DCOP-Net outperforms other state-of-the-art methods. The results of the comparative experiments and ablation studies demonstrate the effectiveness and superiority of our method.

The rest of this article is organized as follows. Section 2 presents the related work. Section 3 describes the proposed methods in detail. Section 4 evaluates the proposed model on three public datasets and provides experimental results. Finally, Section 5 summarizes the entire article and suggests directions for future work.

## 2. Related Work

### 2.1. Few-Shot Medical Image Segmentation

The automatic medical image segmentation models usually adopt visual transformers [28], multi-scale fusion [29], and other methods [30,31] to achieve more accurate, flexible, and reliable results than manual segmentation. However, if large annotated medical image datasets are not available during training, it becomes challenging for large-scale image segmentation models to achieve the best possible results. Unlike natural images, acquiring medical images is more costly, and the annotation process is complex. Few-shot image segmentation provides an effective solution to these issues. Current FSMIS models are mainly divided into methods based on prototype networks [11,12,13,14,15] and interaction-based models [16,17,18,19,20].

As a pioneer in attention-based interactive models for FSMIS, SE-Net [16] achieves effective interactions between the conditioner arm and segmenter arm [32] by introducing channel squeezing and spatial excitation blocks. SSL-ALPNet [13], proposed by Ouyang et al., is the first prototype-based FSMIS method, which can adaptively generate local prototypes according to organ size and predict the FG or BG regions in the query image by distinguishing between FG and BG prototypes. Ouyang et al. [13] also proposed a superpixel-based self-supervised method to meet the demand for labeled data during training. ADNet [25] builds upon this by extending superpixels to supervoxels, fully utilizing the 3D information of the image, and introducing a fixed threshold to segment the query mask. ADNet++ [33] further increases the measurement of predictive uncertainty and proposes a one-step multi-class medical image segmentation framework. CRAP-Net [20] designs a recurrent similarity attention module to preserve the spatial connections between support and query images. CAT-Net [23] limits the attention range while mining the relevance between support and query images.

However, the above works either do not learn the FG information of the query image, or there is a BG mismatch issue at the time of learning. These reasons amplify the prototype bias problem, resulting in inaccurate segmentation of the query images by the model. Taking AD-Net [25] as the baseline network, we propose a DFCA module, which ensures effective interactions between support and query FG features by filtering on BG elements, thereby addressing the BG mismatch issue. It consists of a prior mask generation (PMG) module and a filter cross attention (FCA) module. The PMG module creates a prior mask for the query image, serving as the first barrier to prevent interactions between the query BG and support FG; the FCA module, on the other hand, acts as the second barrier by limiting the impact of low-quality attention scores. This design not only enhances the representation ability of the support prototypes, but also avoids entanglement between the query BG and support FG features.

### 2.2. Attention Mechanism

Some recent attention-based works [34,35] have proved that the attention mechanism can achieve significant improvements in computer vision tasks. Therefore, many few-shot segmentation models [20,23,36,37,38,39] employ cross attention to solve the problem of some features in the query image not appearing in the support features in few-shot segmentation tasks. Particularly, CRAP-Net [20] introduces a cycle-resemblance attention network, facilitating the reciprocal interaction between support and query feature sets. This interaction enables the generation of enhanced support and query features, which are then utilized to refine and adjust their respective informational content.

Although these works have shown promising results, the query BG features are inevitably fused with the unmatched supporting FG features, resulting in feature deviations. Therefore, in this work, we design a dual-filter cross attention (DFCA) module to reduce the entanglement between FG and BG features, thereby improving the quality of the prototype.

### 2.3. Prototype Extraction Method

In FSMIS models, utilizing the similarity between prototypes and query features for segmentation is a mainstream approach. Therefore, enhancing the quality of prototypes becomes a crucial step in improving model performance. Current prototype-based methods may have prototype bias issues [24], which researchers typically address by enhancing the representation ability of individual prototypes or generating multiple prototypes. For instance, PA-Net [27] enhances the representation ability of prototypes through a prototype alignment regularization task. Although single-prototype methods have strong interpretability, they still lack representative details; multi-prototype methods, on the other hand, make up for this deficiency, such as ALPNet [13], which adaptively generates multiple local prototypes based on organ size to supplement the details in the prototype set. Despite this, while multi-prototype methods can provide rich and detailed information, they may also lead to feature fragmentation, making it difficult for a single prototype to express complete organ features.

Inspired by the layer-wrapping of onions in nature, this work proposes the OP module. In order to combine the merits of multiple prototypes and single prototype approaches, we utilize the OP module to retain support image context information to generate multiple prototypes. Subsequently, we aggregate these prototypes into one, enhancing the prototype’s representational capacity and interpretability.

### 2.4. Regularization

Regularization techniques are crucial in improving few-shot segmentation models’ segmentation and generalization capabilities. Previous works [14,15] typically follow this process: utilizing support images and masks to obtain support prototypes and then utilizing these prototypes to segment query images. However, this workflow only partially leverages support images, query images, and masks. Thus, PA-Net [27] proposes a prototype alignment regularization that enhances the model’s generalization ability by segmenting the support images in reverse using the query and predicted masks as new support images and masks to obtain the loss function. In addition, Wang et al. [40] noticed that existing methods neglected supervision of the support images and proposed self-reference regularization, which enhances the support FG prototype’s representational capacity for the FG by segmenting the support images using the support prototypes to obtain the loss function.

However, the above methods still lack in-depth learning and understanding of query images. Therefore, we design QSR, a loss function whose influence gradually increases with the number of training rounds. It can assist in enhancing the model’s comprehension and segmentation of query images.

## 3. Proposed Method

### 3.1. Problem Definition

The FSMIS task aims to use techniques such as meta-learning [41] to train a model, which is trained from the dataset $D_{base}$ containing visible categories $C_{base}$, to quickly generalize to the segmentation of unseen categories $C_{novel}$. This means that $C_{base}\cap C_{novel}=\emptyset$. During the training stage, we follow the episode training method [27], which is commonly used in FSMIS tasks. Specifically, in each *N*-way *K*-shot task, we adopt a random sampling strategy to divide the dataset with novel categories $D_{novel}=\left\{\left(S,Q\right)\right\}$ into a support set $S={\left\{\left(I_{s}^{i},M_{s}^{i}\right)\right\}}_{i=1}^{K}$ and a query set $Q={\left\{\left(I_{q}^{i},M_{q}^{i}\right)\right\}}_{i=1}^{N_{q}}$, where $D_{novel}$ has *N* categories and superscripts *K* and $N_{q}$ are the number of image–mask pairs for the support and query sets, respectively. Moreover, each image–mask pair $\left(I_{s}^{i},M_{s}^{i}\right)$ in the task forms an episode, where $I_{s}^{i}$ and $M_{s}^{i}$ denote the *i*-th support image and its corresponding mask. The subscripts *s* and *q* represent that the categories of images are support or query images, respectively. In general, the inputs to the model are the given support image with its corresponding mask and the query image, while the output is the predicted mask $M_{q}^{pred}$ for the query image.

### 3.2. Architecture Overview

**Figure 1:**
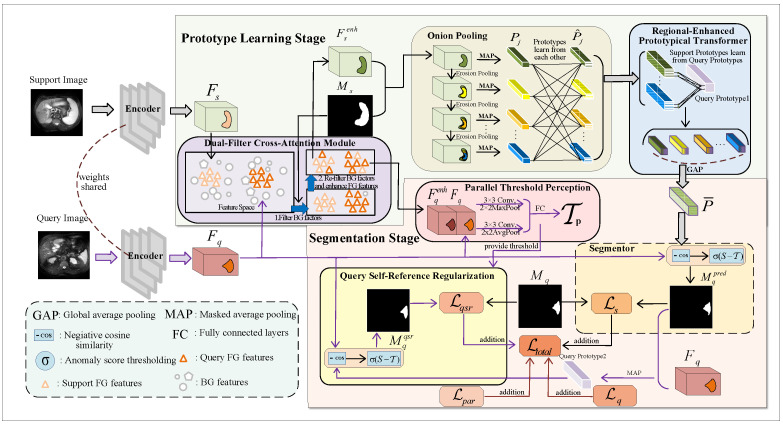
Overview of the proposed DCOP-Net architecture. This model consists of two stages, i.e., the prototype learning stage and the segmentation stage, which are used to complete the prototype learning and the segmentation of the query image, respectively.

As shown in Figure 1, the proposed DCOP-Net’s workflow is divided into two main stages: prototype learning and segmentation. The prototype learning stage, composed of the DFCA, OP, and Regional-enhanced Prototypical Transformer (RPT) modules [42], has the core task of learning high-quality support prototypes. Meanwhile, the segmentation stage, which consists of the PTP, Segmentor, and QSR modules, is primarily responsible for segmenting the query images.

Specifically, we first input the support and query images into a weight-sharing feature encoder to extract feature maps. Then, in the prototype learning stage, we propose a novel strategy for learning prototypes. In the first step, we input support features and query features into the DFCA module to obtain the enhanced support features and the enhanced query features. The DFCA module filters background features and employs a cross-attention-based approach, enabling the support FG features to learn and integrate the query FG features, effectively. In the second step, we input the enhanced support features from above and their ground truth masks into the OP and RPT modules to obtain the support prototype. The innovative OP module can generate onion-layer masks by eroding support mask boundaries layer by layer and then generate multiple prototypes using MAP. Afterward, the OP and RPT modules enable the prototypes to learn from each other, thus enhancing their representation ability for FG features. Finally, we employ global average pooling (GAP) to aggregate multiple prototypes into one.

During the segmentation stage, we utilize the segmentation method based on anomaly scores [25] to segment query image and design the QSR module to enhance the model’s generalization ability. Specifically, we concatenate the query features output by the DFCA module with the original query features and then input the result into the parallel-structured PTP module for feature processing, generating parameters for thresholding the anomaly scores in the subsequent context. In the subsequent Segmentor module, we utilize the query features and the prototype extracted from the prototype learning stage to calculate the negative cosine similarity (i.e., anomaly scores) [25]. Then, we employ the parameters output by the PTP module to binarize calculation results, thereby obtaining the predicted mask. Significantly, after completing segmentation, we propose a QSR module that resegments the query image using the query image and the predicted mask. Thus, QSR creates a feedback mechanism regarding the query image, enhancing the accuracy and consistency of the model when processing query images.

### 3.3. Prototype Learning Stages

Before the prototype learning stage, we employ a parameter-sharing feature encoder $f_{\theta }\left(\cdot \right)$ [43] to extract feature vectors from support image $I_{s}$ and query image $I_{q}$. This process can be represented as follows:

(1) $$F_{s}=f_{\theta }\left(I_{s}\right)$$

(2) $$F_{q}=f_{\theta }\left(I_{q}\right)$$

where $F_{s}$ and $F_{q}$ are the support and query features, respectively.

#### 3.3.1. Dual-Filter Cross Attention Module

During the prototype learning stage, many FSMIS models leverage cross-attention-based approaches [22,23] to achieve the interactions between support and query features, enhancing support FG features’ ability to represent FG classes. However, feature interactions that fail to filter BG features may have the query BG incorrectly fused with the support FG features, which can directly lead to lower prototype quality and indirectly lower segmentation accuracy.

To address this problem, we restrict the interaction between query FG features and support FG features by purposefully filtering BG features with the proposed DFCA module. This design allows for the stable fusion of FG features, thereby significantly enhancing the representation of the support FG features while avoiding BG mismatching issue [44].

**Figure 2:**
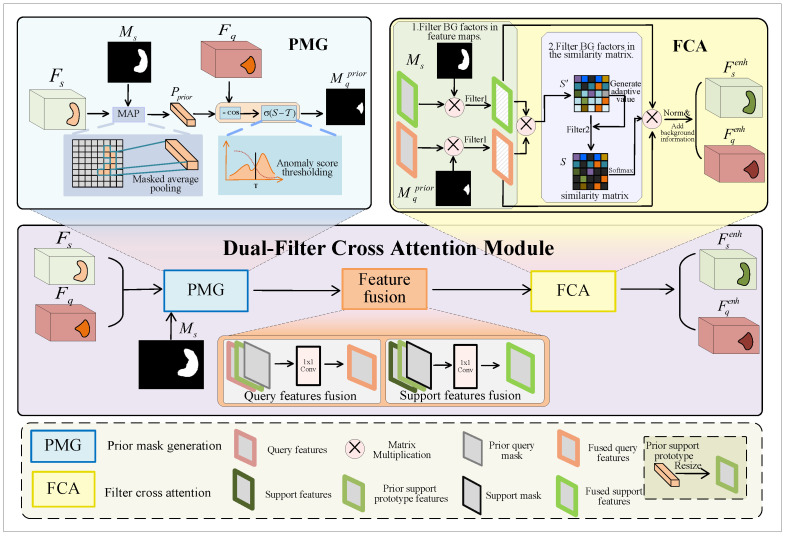
Dual-filter cross attention module. This module takes support features, query features, and the support mask as inputs and outputs enhanced support features and enhanced query features after three blocks of PMG, feature fusion, and FCA.

The entire process is illustrated in Figure 2. Firstly, we input the support features, query features, and support mask into the prior mask generation (PMG) block to obtain a prior query mask. Then, to reduce the disparity between the FG features of support and query images, we fuse the support features $F_{s}$, support mask $M_{s}$ (or query features $F_{q}$, prior query mask $M_{q}^{prior}$), and prior support prototype map $F_{p}$ using 1 × 1 convolution, outputting the fused support features $F_{s}^{fused}$ (or fused query features $F_{q}^{fused}$). Finally, we employ the proposed filter cross attention (FCA) block to facilitate mutual learning among the FG features while filtering out the influence of BG factors as much as possible, resulting in enhanced support features $F_{s}^{enh}$ (or enhanced query features $F_{q}^{enh}$).

(1) Prior mask generation

The PMG module applies MAP to the support image and support mask to compute the prior support prototype. The prior support prototype is then used to calculate the cosine similarity with the query image, which is subsequently thresholded to obtain the prior query mask. Using the generated prior query mask, we can initially separate the FG and BG regions of the query image.

Specifically, as shown in Figure 2, we conduct MAP [24] to obtain a prior support prototype $P_{prior}$ by leveraging the support features $F_{s}\left(h,w\right)$ and corresponding mask $M_{s}\left(h,w\right)$. The mathematical form of this process is expressed as follows:

(3) $$P_{prior}=\frac{\sum_{h,w}F_{s}\left(h,w\right)⊙M_{s}\left(h,w\right)}{\sum_{h,w}M_{s}\left(h,w\right)}$$

where ⊙ represents the Hadamard product, (h,w) denotes the pixel position in the original mask, $F_{s}\left(h,w\right)$ and $M_{s}\left(h,w\right)$ represent the support features and the corresponding binary FG mask, respectively, and $P_{prior}$ represents the support prototype in the process of generating the prior mask.

Subsequently, we utilize $P_{prior}$ to compute the negative cosine similarity (i.e., anomaly scores) with each location in $F_{q}\left(h,w\right)$. This can be denoted as:

(4) $$S_{prior}\left(h,w\right)=-\alpha \frac{F_{q}\left(h,w\right)\cdot P_{prior}}{∥F_{q}\left(h,w\right)∥∥P_{prior}∥}$$

where $S_{prior}\left(h,w\right)$ is the anomaly score map for each position in $F_{q}\left(h,w\right)$, $F_{q}\left(h,w\right)$ represents the query features, α is the scaling factor introduced by Oreshkin et al. [45] to facilitate backpropagation, which is generally set to 20, and $∥\cdot ∥$ represents the norm of a matrix.

After that, to make the process of thresholding the anomaly scores differentiable, we employ a shifted Sigmoid function [25], which ensures that regions with anomaly scores below the prior threshold $T_{a}$ obtain a higher FG probability, thereby obtaining the final prior query mask $M_{q}^{prior}$. $T_{a}$ is typically set to −10.0 [25]. The entire process is illustrated in the following formula:

(5) $$M_{q}^{prior}=1-\sigma \left(S_{prior}\left(h,w\right)-T_{a}\right)$$

where $\sigma \left(\cdot \right)\;$ is the Sigmoid function with a steepness parameter k=0.5.

(2) Feature fusion

Before the support and query FG features interact, it is important to recognize that they may not be similar. In order to close their gaps and improve the quality of interaction, we fuse the query features $F_{q}$, prior support prototype features $F_{p}$, and prior query mask $M_{q}^{prior}$. Moreover, we fuse the support features $F_{s}$, prior support prototype features $F_{p}$, and support mask $M_{s}$. This aligns the query features with the support features in the same feature space, reducing the distribution discrepancy between them.

Specifically, as shown in Figure 2, we process $P_{prior}$ to match the size of $F_{s}$, thereby obtaining prior support prototype features $F_{p}$. Subsequently, we conduct channel concatenation on $F_{q}$, $F_{p}$, and $M_{q}^{prior}$ and use 1×1 convolution for dimensionality reduction to achieve feature fusion. Similarly, $F_{s}$ will be concatenated with $F_{p}$ and $M_{s}$ to achieve feature fusion. The fused query features $F_{q}^{fused}$ and fused support features $F_{s}^{fused}$ are calculated as:

(6) $$F_{q}^{fused}=Conv_{1\times 1}\left(Concat\left(F_{q},F_{p},M_{q}^{prior}\right)\right)$$

(7) $$F_{s}^{fused}=Conv_{1\times 1}\left(Concat\left(F_{s},F_{p},M_{s}\right)\right)$$

where Concat(·) denotes channel concatenation and $Conv_{1\times 1}$ is 1×1 convolution operation.

(3) Filter cross attention

As shown in Figure 2, after feature fusion, FCA takes the fused support features $F_{s}^{fused}$, fused query features $F_{q}^{fused}$, and both corresponding masks as inputs and then outputs the enhanced support features $F_{s}^{enh}$ and enhanced query features $F_{q}^{enh}$. We use masks as the first filter to filter BG factors in feature maps, which involves performing a Hadamard product between masks and features. This process is shown in the following formulas:

(8) $$\begin{array}{c} F_{s}^{fg}=M_{s}⊙F_{s}^{fused} \end{array}$$

(9) $$\begin{array}{c} F_{q}^{fg}=M_{q}^{prior}⊙F_{q}^{fused} \end{array}$$

where $F_{s}^{fg}$ and $F_{q}^{fg}$ are the FG parts of fused support features and fused query features, respectively.

Then, we perform a cross-attention-based approach [20,23] to obtain $F_{s}^{enh}$ and $F_{q}^{enh}$. We take obtaining $F_{s}^{enh}$ as an example to illustrate the whole process. FCA first projects support FG features into a sequence $Q_{s}$ and then projects the query FG features into sequences $K_{s}\&V_{s}$.

(10) $$Q_{s}=W_{Q_{s}}F_{q}^{fg}+B_{Q_{s}}$$

(11) $$K_{s}=W_{K_{s}}F_{q}^{fg}+B_{K_{s}}$$

(12) $$V_{s}=W_{V_{s}}F_{q}^{fg}+B_{V_{s}}$$

where $W_{Q_{s}}$ and $B_{Q_{s}}$ are weight matrices and bias terms for generating $Q_{s}$, $W_{K_{s}}$ and $B_{K_{s}}$ are weight matrices and bias terms for generating $K_{s}$, and $W_{V_{s}}$ and $B_{V_{s}}$ are weight matrices and bias terms for generating $V_{s}$. After that, FCA conducts matrix multiplication to calculate the similarity between $Q_{s}$ and $K_{s}$ to obtain the similarity matrix $S_{m}^{\prime }$:

(13) $$\begin{array}{c} S_{m}^{\prime }=\frac{Q_{s}\cdot K_{s}^{T}}{\sqrt{d_{k}}} \end{array}$$

where $d_{k}$ is the dimension of $K_{s}$.

However, considering the inaccuracy of the prior query mask, the first filter may not completely filter BG factors. We design an filtering function in cross attention as the second filter to filter BG factors in $S_{m}^{\prime }$:

(14) $$\begin{array}{c} Filter\left(S_{m}^{\prime }\right)=\left\{\begin{array}{c} S_{m},\;S_{m}^{\prime }>V_{filter}\\ -\infty ,\;otherwise \end{array}\right.,\\ V_{filter}=\left(max\left(S_{m}^{\prime }\right)+mean\left(S_{m}^{\prime }\right)\right)/2 \end{array}$$

where Filter(·) is the filtering function, $S_{m}$ is the filtered similarity matrix, $V_{filter}$ is the adaptive value for filtering BG factors, and max(·) and mean(·) represent the operations of obtaining the maximum value and the average value, respectively.

The filtering function adaptively filters low-quality similarity scores that may exist in $S_{m}^{\prime }$. Then, we use the softmax function to normalize $S_{m}$ and fuse the result with $V_{s}$, which can be denoted as:

(15) $${\hat{F}}_{s}^{fg}=softmax\left(S_{m}\right)\cdot V_{s}$$

where ${\hat{F}}_{s}^{fg}$ is the FG regions of enhanced support features.

After that, FCA adds the corresponding BG information to ${\hat{F}}_{s}^{fg}$, yielding the enhanced support features $F_{s}^{enh}$:

(16) $$F_{s}^{enh}=add_{bg}\left({\hat{F}}_{s}^{fg}\right)$$

where $add_{bg}\left(\cdot \right)$ represents the operation of adding BG information.

Similarly, we can obtain the enhanced query features $F_{q}^{enh}$. The whole process is as follows:

(17) $$F_{q}^{enh}=add_{bg}\left(softmax\left(Filter\left(\frac{Q_{q}\cdot K_{q}^{T}}{\sqrt{d_{k}}}\right)\right)\cdot V_{q}\right)$$

where sequence $Q_{q}$ is the query FG features being projected and sequences $K_{q}\&V_{q}$ are the support FG features being projected.

#### 3.3.2. Onion Pooling

During the prototype extraction, prototype bias represents a significant challenge that every prototype learning method must confront. In addition, due to intra-class diversity, there will be significant differences between support and query features, so the intra-class bias problem is also a significant challenge.

To address the above challenges, we propose the OP module and introduce RPT module. We use the erosion pooling (EP) operation to help prototypes acquire richer contextual information, thus enhancing the ability of prototypes to represent FG features and alleviating the problem of prototype bias. Furthermore, we alleviate the intra-class bias by using the self-attention mechanism and the RPT module to reduce the inconsistency between prototypes.

**Figure 3:**
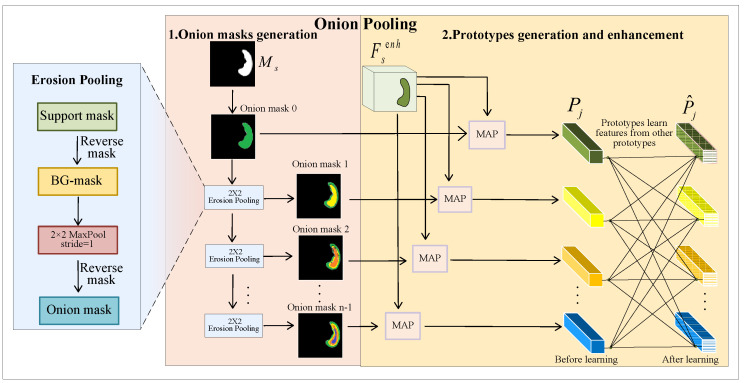
Onion pooling module. This module extracts multiple support prototypes by conducting erosion pooling (EP), masked average pooling (MAP), and a self-attention-based approach on the support mask and the enhanced support features.

Specifically, we use the proposed EP operation to erode the support mask, creating layer-by-layer onion masks that prepare for extracting prototypes. Then, we conduct the MAP method to generate prototypes. Finally, we employ a self-attention-based method to enable mutual learning within the prototypes. This process is illustrated in Figure 3.

(1) Onion mask generation

Here, we refer to the eroded support masks as onion masks. We conduct several EP operations to shrink the FG region of the support mask progressively, resulting in multiple onion masks.

In the beginning, we obtain the BG mask by reversing the support mask. Subsequently, we perform a 2×2 max-pooling operation on the BG mask to reduce the size of the FG region. These two steps simultaneously expand the background region of the mask while reducing the foreground region. Finally, we reverse the pooled BG mask again to obtain the FG mask, which is the onion mask. This process can be expressed as follows:

(18) $${\overset{⌢}{M}}_{s}^{j}\left(h,w\right)=EP_{2\times 2}^{j}\left({\overset{⌢}{M}}_{s}^{j-1}\left(h,w\right)\right)$$

where ${\overset{⌢}{M}}_{s}^{j}\left(h,w\right)$ represents the onion mask obtained after *j*-th (j≥1) erosion pooling. It is worth noting that $M_{s}^{j-1}\left(h,w\right)$ is support mask $M_{s}\left(h,w\right)$ when j=1. $EP_{2\times 2}^{j}\left(\cdot \right)$ represents the EP operation using a 2×2 window.

Finally, we obtain *n* onion masks by executing EP *n* — 1 times. Empirically, the upper limit $N_{max}$ of the number of onion layers is typically set to 4.

(2) Prototypes generation and enhancement

After obtaining onion masks, we conduct MAP to extract support prototypes by leveraging enhanced support features and onion masks:

(19) $$P_{j}=\frac{\sum_{h,w}F_{s}^{enh}\left(h,w\right)⊙{\overset{⌢}{M}}_{s}^{j}\left(h,w\right)}{\sum_{h,w}{\overset{⌢}{M}}_{s}^{j}\left(h,w\right)}$$

where $P_{j}$ represents the support prototype extracted using the *j*-th onion mask. It is worth mentioning that we obtain *n* support prototypes from the *n* generated onion masks.

To alleviate intra-class bias, we employ a self-attention mechanism [46] to enable prototypes to learn from each other internally. Precisely, we concatenate the prototypes into one, then project the result into the sequences $Q^{\prime }$, $K^{\prime }$, and $V^{\prime }$:

(20) $$Q^{\prime }=W_{Q^{\prime }}\cdot Concat\left(P_{1},P_{2},\cdots ,P_{n}\right)+B_{Q^{\prime }}$$

(21) $$K^{\prime }=W_{K^{\prime }}\cdot Concat\left(P_{1},P_{2},\cdots ,P_{n}\right)+B_{K^{\prime }}$$

(22) $$V^{\prime }=W_{V^{\prime }}\cdot Concat\left(P_{1},P_{2},\cdots ,P_{n}\right)+B_{V^{\prime }}$$

where $W_{Q^{\prime }}$, $W_{K^{\prime }}$, $W_{V^{\prime }}$ are weight matrices for generating $Q^{\prime }$, $K^{\prime }$, $V^{\prime }$, respectively. $B_{Q^{\prime }}$, $B_{K^{\prime }}$, and $B_{V^{\prime }}$ are bias terms for generating $Q^{\prime }$, $K^{\prime }$, and $V^{\prime }$, respectively. Subsequently, we compute the dot product similarity between $Q^{\prime }$ and $K^{\prime }$, then normalize it using a softmax function to obtain the attention scores. We employ the attention scores to weight $V^{\prime }$ and then split the result into *n* prototypes. The entire process is illustrated as follows:

(23) $$\left[{\hat{P}}_{1},{\hat{P}}_{2},\cdots ,{\hat{P}}_{n}\right]=split\left(softmax\left(\frac{Q^{\prime }K^{\prime T}}{\sqrt{d_{k}}}\right)V^{\prime },n\right)$$

where ${\hat{P}}_{j}$ represents the support prototypes generated by mutual learning through the self-attention method (where 1≤j≤n) and split(·,n) represents splitting the concatenated prototype into *n* prototypes.

#### 3.3.3. Regional-Enhanced Prototypical Transformer Module

After extracting the prototypes using the OP module, we introduce the RPT module to mitigate the effects of intra-class bias. As shown in Figure 1, the RPT module employs the methods presented in PRNet [47] to extract a coarse query prototype from $F_{q}$. Subsequently, it utilizes this query prototype to refine support prototypes. In the end, GAP operation is used to regenerate the optimal global prototype $\bar{P}$. The whole process is shown below.

(24) $$\bar{P}=GAP\left(RPT\left(\left[{\hat{P}}_{1},{\hat{P}}_{2},\cdots ,{\hat{P}}_{n}\right],F_{q}\right)\right)$$

where GAP(·) is the global average pooling operation and RPT(·) is the operation using the RPT module.

In the next segmentation stage, $\bar{P}$ is used to segment the query image.

### 3.4. Segmentation Stages

#### 3.4.1. Parallel Threshold Perception Module

Few-shot medical image segmentation models typically make predictions by measuring the similarity between support and query images, followed by thresholding the prediction results with a parameter to generate the final segmentation. In AD-Net [25], Hansen et al. proposed a learnable threshold during training, combined with a Sigmoid function with a steepness parameter of 0.5, to assign higher foreground probabilities to regions with values below the threshold T and lower foreground probabilities to regions above T.

In the standard segmentation setting, test-class slices are accessible during training, enabling the learned threshold for anomaly detection to be optimized on the segmentation targets. However, if test-class slices are excluded during training, the test classes become entirely unseen categories for the model. Consequently, the threshold, not being optimized on these segmentation targets, fails to adapt directly to such categories. This limitation may explain the suboptimal performance of AD-Net in the Setting 2. Therefore, it is essential to dynamically generate thresholds based on the content of the query image, rather than relying solely on the loss between prediction results and ground truth to learn the threshold.

Accordingly, to address this issue, we propose the PTP module to enhance the robustness and efficacy of the threshold. This module processes $F_{q}$ and $F_{q}^{enh}$ through a dual-path structure to fully extract features and reduce the effect of noise. Then, after downsampling, the module outputs the threshold $T_{p}$ through the fully connected layer.

**Figure 4:**
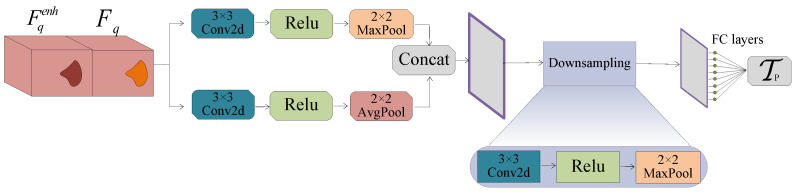
Parallel threshold perception module. This module inputs query features and enhanced query features and outputs a robust and effective threshold after dual-path feature processing and establishing a fully connected layer.

In detail, Figure 4 shows the dual-path structure. One branch retains the salient features of the original image through 3×3 convolution and 2×2 max-pooling operations, and the other retains the image’s overall features by combining 3×3 convolution and 2×2 avg-pooling operations. These two complementary branches help the module extract both salient and global features effectively, reducing noise interference.

(25) $$F_{q}^{1}=MaxPool_{2\times 2}\left(Relu\left(Conv_{3\times 3}\left(F_{q},F_{q}^{enh}\right)\right)\right)$$

(26) $$F_{q}^{2}=AvgPool_{2\times 2}\left(Relu\left(Conv_{3\times 3}\left(F_{q},F_{q}^{enh}\right)\right)\right)$$

where $F_{q}^{1}$ and $F_{q}^{2}$ represent the feature maps obtained by the first branch and the second one, respectively. Relu(·) is a commonly used activation function in few-shot learning. $Conv_{3\times 3}\left(\cdot \right)$ is a 3×3 convolution operation.

Then, PTP concatenates the outputs of the two branches. To resize the concatenated features to match the fully connected layer, we downsample them using 3×3 convolution and 2×2 max-pooling. Finally, the threshold $T_{p}$ is predicted through the fully connected layer:

(27) $$T_{p}=FC\left(DS\left(Concat\left(F_{q}^{1},F_{q}^{2}\right)\right)\right)$$

where FC(·) is the operation of using fully connected layer and DS(·) is the downsampling operation, which means performing 2×2 convolution, Relu(·), and 2×2 max-pooling in sequence.

#### 3.4.2. Segmentor

After obtaining $T_{p}$, we employ the anomaly-score-based segmentation method [25] to obtain the predicted mask for query image $M_{q}^{pred}$. As shown in Figure 1, Segmentor calculates the negative cosine similarity between the prototype $\bar{P}$ and the query features $F_{q}$, yielding anomaly scores for each position $\left(h,w\right)$ of the query features:

(28) $$S_{pred}\left(h,w\right)=-\alpha \frac{F_{q}\left(h,w\right)\cdot \bar{P}}{∥F_{q}\left(h,w\right)∥∥\bar{P}∥}$$

where $S_{pred}\left(h,w\right)$ is the predicted anomaly score map for $F_{q}$ and α is the same scaling factor as in Equation (4).

Then, we perform soft thresholding using $T_{p}$, thereby generating the predicted query mask $M_{q}^{pred}$. The process is depicted as follows:

(29) $$M_{q}^{pred}=1-\sigma \left(S_{pred}\left(h,w\right)-T_{p}\right)$$

where $M_{q}^{pred}$ is the predicted mask for query image and σ is the same Sigmoid function as in Equation (5).

#### 3.4.3. Query Self-Reference Regularization

Existing FSMIS segmentation models [13,14] usually use the similarity between the support and query images to obtain the predicted mask. It is evident that existing models primarily focus on the information stream from support features to query features, overlooking the supervisory role of the query image itself during the segmentation process.

To enhance the model’s ability to generalize and refine its comprehension and segmentation capabilities for query images, we propose query self-reference regularization (QSR). It allows the information from the query image to be streamed back into itself, effectively reinforcing the model’s generalization performance. QSR is inspired by the assumption that the more accurately a mask labels an image, the better the model can learn and segment FG features. Suppose that the model generates a more accurate predicted query mask as training rounds increase. In this case, we use the predicted query mask as a ‘new support mask’ and the query image as a ‘new support image’ to resegment the query image. After that, we can use cross-entropy loss to measure the prediction mask and the ground truth mask.

Specifically, QSR initially conducts MAP operation, utilizing $F_{q}$ and $M_{q}^{pred}$ as inputs to extract the query prototype. Subsequently, QSR calculates the negative cosine similarity between the query prototype and the query features, then thresholds the calculation results using $T_{p}$ to finalize another predicted query mask $M_{q}^{qsr}$. We can perform cross-entropy loss on the prediction result and the query mask. In addition, we introduce a growth factor β for QSR loss, which can increase with the number of training times to gradually amplify the influence of QSR loss. The QSR loss is represented as follows:

(30) $$L_{qsr}=\frac{1}{HW}\beta \sum_{h,w}\left\{\left(1-M_{q}\right)\log\left(1-M_{q}^{qsr}\right)+M_{q}\log M_{q}^{qsr}\right\}$$

where $L_{qsr}$ is the query self-reference regularization loss, HW is the total number of points in the spatial location, β is the growth factor, which is initially set to 0.02 and increases by 0.02 with each epoch, and $M_{q}^{qsr}$ is the query prediction mask generated during the query self-reference regularization process.

#### 3.4.4. Loss Function

The loss function can guide FSMIS models’ learning and enhance training efficiency. Here, we adopt the cross-entropy loss function to measure the dissimilarity between the predicted mask $M_{q}^{pred}$ and the ground truth mask $M_{q}$ for the query image. This process is mathematically shown below:

(31) $$\;L_{s}=-\frac{1}{HW}\sum_{h,w}\left\{\left(1-M_{q}\right)\log\left(1-M_{q}^{pred}\right)+M_{q}\log M_{q}^{pred}\right\}$$

where $L_{s}$ is the binary cross-entropy segmentation loss and $M_{q}$ is the query ground truth mask.

Additionally, as is common with other FSMIS models [27,42], we also add query loss and prototype alignment regularization loss to the overall loss function, as depicted in the subsequent formula:

(32) $$\;L_{q}=-\frac{1}{HW}\sum_{h,w}\left\{\left(1-M_{q}\right)\log\left(1-M_{q}^{query}\right)+M_{q}\log M_{q}^{query}\right\}$$

(33) $$L_{par}=-\frac{1}{HW}\sum_{h,w}\left\{\left(1-M_{s}\right)\log\left(1-M_{s}^{par}\right)+M_{s}\log M_{s}^{par}\right\}$$

where $L_{q}$ is the query loss, $M_{q}^{query}$ is the predicted mask for the query image generated during the calculation of the query loss, $L_{par}$ is the prototype alignment regularization loss, and $M_{s}^{par}$ is a predicted mask for the support image generated during the calculation of the prototype alignment regularization loss.

In summary, the overall loss function is given by the following formula:

(34) $$L_{total}=L_{s}+L_{q}+L_{par}+L_{qsr}$$

where $L_{total}$ is the overall loss function.

## 4. Experiment

### 4.1. Datasets

We evaluate the proposed method on three publicly available datasets:

(1) CHAOS-MRI [48] is an abdominal MRI dataset from the ISBI 2019 Combined Healthy Abdominal Organ Segmentation Challenge. The dataset comprises 20 3D T2-SPIR MRI scans containing approximately 36 slices. Among them, we selected the left kidney (LK), right kidney (RK), liver, and spleen for the assessment.

(2) Synapse-CT [49] is an abdominal CT dataset obtained from the MICCAI 2015 Multi-Atlas Abdomen Labeling Challenge. It consists of 30 3D abdominal CT scans, and we likewise selected the left kidney, right kidney, liver, and spleen.

(3) CMR [50] is a cardiac MRI dataset from the MICCAI 2019 Multi-Sequence Cardiac MRI Segmentation Challenge. It contains 35 3D cardiac MRI scans, each divided into approximately 13 slices. We selected the blood pool (LV-BP), left ventricle myocardium (LV-MYO), and right ventricle myocardium (RV).

### 4.2. Experimental Settings and Evaluation Metric

The current evaluation methods for FSMIS task are generally aligned with ALPNet [13]. In order to make a fair comparison, we also adopt the same experimental settings.

(1) Setting 1 allows FG category organs in the input image to appear in the background. This situation means that the new category used for evaluation is not unseen.

(2) Setting 2 does not allow FG category organs in the input image to appear in any form. This situation means that the new category used for evaluation is unseen. It is worth stating that Setting 2 cannot be applied to CMR dataset due to the difficulty of excluding slices containing the target class from the cardiac dataset. In contrast, there is no such difficulty in MRI and CT datasets.

Before training, we process the training dataset employing the same data preprocessing technique used in ALP-Net [10], where 2D slices of 3D scans are formatted as 256×256 and replicated three times along the channel dimensions to fit the model’s input. For model training, we adopt the self-supervision method based on supervoxels used in AD-Net [25] to train the model.

For evaluating the model output, we adopt the Dice score, which is commonly used in FSMIS tasks, as an evaluation metric. The Dice score [10] measures the similarity between the predicted mask and the ground truth based on the pixel-level overlap of the images. For ground truth *A* and predicted mask *B*, their Dice score can be expressed as:

(35) $$D\left(A,B\right)=2\frac{\left|A\cap B\right|}{\left|A\right|+\left|B\right|}\times 100\%$$

where the value of D(A,B) ranges between 0 and 1. When the value equals 1, it means the segmentation result matches precisely. When the value equals 0, it means a complete disjunction of the segmentation result and the actual annotation. Thus, the higher the value of the Dice score, the better the segmentation result.

### 4.3. Implementation Details

In DCOP-Net, the encoder is a pretrained ResNet-101 network [43] on the MS-COCO dataset [51]. For the training of our model, we set the total number of required iterations to 15K, comprising 5K iterations per epoch. Moreover, the initial learning rate is set to 0.001, the batch size to 1, and the decay rate to 0.98. Training takes 2.25 h on an Nvidia RTX 4060 GPU. In order to simulate the scarcity of data in healthcare scenarios, the experiments follow the 1-way 1-shot task. In order to avoid the influence of the dataset on the experimental results, we adopt the five-fold cross-validation [25] to record the results.

### 4.4. Results and Comparison with SOTA Methods

In order to determine the effectiveness and superiority of DCOP-Net, we compare our model with FSMIS models, including ALP-Net [10], SE-Net [16], AAS-DCL [19], CRAP-Net [20], CAT-Net [23], AD-Net [25], Q-Net [26], PA-Net [27], SR&CL [40], RPT-Net [42], DSP-Net [52], GMRD [14], and PAMI [53]. Table 1 presents the experimental results of the currently proposed FSMIS models on the Synapse-CT and CHAOS-MRI datasets under Setting 1 and Setting 2. Table 2 presents the experimental results on the CMR dataset under Setting 1.

As shown in Table 1, the average Dice scores of DCOP-Net in the three scenarios are generally better than the other methods. Specifically, under Setting 1, our model’s average Dice score on the CHAOS-MRI dataset is 82.64%, which exceeds the highest current result (RPT-Net) by 0.2%. Especially for the segmentation of spleen organs, our method outperforms the highest current result (RPT-Net) by 4.15%. Our method achieves an average Dics score of 74.59% on the Synapse-CT dataset. Under Setting 2, DCOP-Net achieves average Dice score of 71.72% and 80.74% on the Synapse-CT and CHAOS-MRI datasets, respectively. In particular, DCOP-Net’s average Dice score on the CHAOS-MRI dataset exceeds the second-best method (PAMI) by 1.21%. Notably, DCOP-Net achieves 4.95%, outperforming the suboptimal method PAMI, and 1.94%, outperforming the suboptimal method RPT-Net, in the segmentation of spleen and LK, respectively, on the CHAOS-MRI dataset. Moreover, comparing the segmentation results of our model on the Synapse-CT dataset under Setting 1, our model outperforms RPT-Net, demonstrating our model’s ability to generalize under more stringent conditions. In addition, compared to processing MRI-modality medical images, few-shot segmentation models exhibit a decrease in overall segmentation accuracy when dealing with lower-contrast CT-modality datasets. Addressing this challenge remains a crucial task for current few-shot medical image segmentation models.

As shown in Table 2, although the average Dice score of our model does not exceed the current best method, it achieves satisfactory results in the segmentation of LV-BP and RV. There are possible reasons for this situation. The poor sample quality of the CMR dataset makes it difficult for the model to adequately learn enough features to segment accurately. Moreover, the shape and structure of LV-MYO are relatively complex, making it difficult to accurately distinguish between the tissues surrounding the LV wall and the myocardial cell wall itself.

**Figure 5:**
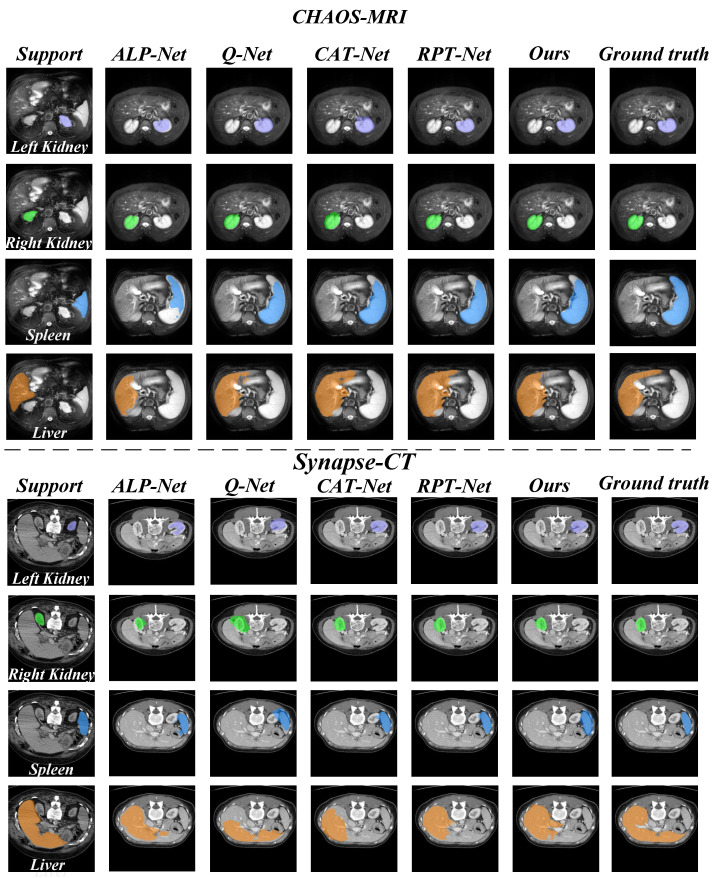
Comparison of qualitative results between our model and other methods. The upper row illustrates the results on the CHAOS-MRI dataset, while the lower row shows the results on the Synapse-CT dataset.

**Figure 6:**
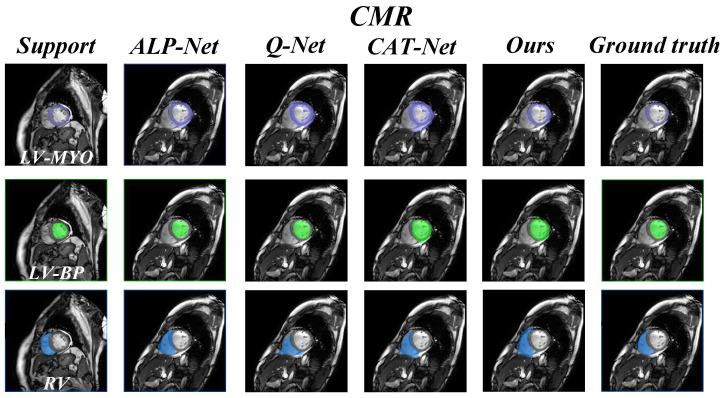
Comparison of qualitative results between our model and other methods on the CMR dataset.

In addition, we also present a visual comparison of the prediction results of existing models on the three datasets in Figure 5 and Figure 6. It can be seen from Figure 5 that our model exhibits fewer over-segmentation and obtains more accurate segmentation results in CHAOS-MRI and Synapse-CT datasets. As shown in Figure 6, our model achieves good results in segmenting LV-BP and RV organs in the CMR dataset.

### 4.5. Ablation Studies

We conduct ablation studies on the CHAOS-MRI dataset under Setting 1 or Setting 2. Specifically, we examine component performance and the validity of the generation of prior masks, extraction of prototypes, and generation of thresholds.

#### 4.5.1. Effect of Each Component

Notably, we utilize AD-Net as Baseline in this part. As shown in Table 3, we conduct experiments with several combinations of the proposed models. For example, “Baseline + DFCA” indicates that the DFCA module is embedded in the Baseline network, while “Baseline + DFCA + OP + PTP + QSR” indicates that DFCA, OP, PTP, and QSR modules are embedded in the Baseline network.

It can be seen from Table 3 that the overall segmentation performance of the model shows a steady improvement as our proposed modules are progressively embedded into the Baseline. Specifically, the overall segmentation performance of the model is improved by 2.06% with the addition of the DFCA module to the Baseline. On this basis, adding OP further improves the overall performance of the Baseline by 1.14% by fusing contextual information in the prototype. The overall performance of the Baseline is improved by 3.2% with the combination of the DFCA module and the OP module, which also shows that the prototype bias problem is effectively mitigated.

Furthermore, to validate the effectiveness of the QSR strategy in enhancing the model’s generalization ability, we conduct additional ablation experiments under Setting 2. As clearly shown in Table 4, when the QSR module is not integrated, the model achieves an average Dice score of 79.96% on the CHAOS-MRI dataset. After incorporating the QSR strategy, the overall segmentation accuracy improves to 80.74%, with noticeable improvements in the segmentation accuracy of the spleen, liver, LK, and RK. Moreover, when evaluated on the Synapse-CT dataset, the model with the QSR strategy achieves an overall segmentation accuracy of 71.86%, surpassing the version without QSR by 0.62%. These results demonstrate that integrating the QSR strategy consistently enhances the model’s segmentation performance on both CHAOS-MRI and Synapse-CT datasets, thereby improving its generalization ability for segmenting unseen categories. Overall, with the gradual integration of the modules, the model’s segmentation performance continues to improve, which validates the effectiveness of our work.

#### 4.5.2. Validity of Different Prior Mask Generation Methods

In order to evaluate the role of PMG, we conduct an ablation study in DCOP-Net. The results in Table 5 clearly and unambiguously show that our model achieves a 0.49% higher average Dice score when applying PMG than when applying mask incorporate feature extraction (MIFE) [23]. This improvement is mainly attributed to the fact that MIFE directly employs the normalized value of cosine distance as the prediction probability, which may introduce more noise. In contrast, we use the shifted Sigmoid function processing to discretize the anomaly score results more obviously, which enhances the model’s ability to recognize image boundaries.

#### 4.5.3. Validity of Different Prototype Extraction Methods

In order to verify the validity of OP in extracting prototypes, we compare it with the single prototype method MAP [24], the multiple prototype methods adaptive local prototype pooling (ALP) [10], and regional prototype generation (RPG) [42] for the ablation study.

**Figure 7:**
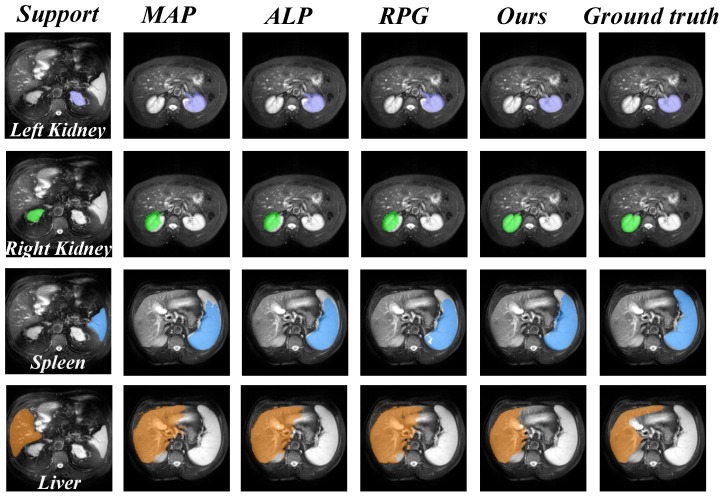
Comparison of validity of prototype extraction methods between our method and other methods on the CHAOS-MRI dataset.

The comparison results are summarized in Table 6 and Figure 7. It can be seen from the results that the overall segmentation of the model is less effective than the ALP, RPG, and OP methods when the single prototype method MAP is used. The reason for this is that in a few-shot environment where it is already difficult to describe the FG categories, MAP may further blur the detailed features, leading to a lower model segmentation accuracy. In contrast, the model achieves 0.52% better results when applying the RPG method than the ALP method. However, RPG divides FG features into dozens of fragmented prototypes while ignoring the collaborative relationships between support prototypes.

In our OP module, we exploit the self-attention mechanism to make support prototypes learn from each other, enhancing the representation ability of support prototypes. Thus, our method achieves better results in segmenting the three organs RK, LK, and spleen than the RPG method and achieves a 0.96% higher overall segmentation accuracy.

#### 4.5.4. Ablation Study on the Maximum Number of Onion Layers

The number of onion layers depends on the size of the target organ, but there is an upper limit $N_{max}$. This $N_{max}$ is a hyperparameter determined experimentally. We conduct experiments using DCOP-Net under Setting 1 on the CHAOS-MRI dataset, and the experimental results are presented in Table 7. As shown in the table, the model achieves the best segmentation performance when $N_{max}=4$, reaching an average Dice score of 82.64%. Therefore, this result is 0.67% higher than the suboptimal case with $N_{max}=5$. This demonstrates that the optimal value for the upper limit of onion layers is 4.

#### 4.5.5. Validity of Different Threshold Generation Methods

In order to verify the effectiveness of PTP, we compare it with the query-informed threshold adaptation (TA) method in Q-net [26]. Table 8 illustrates the comparison results. As can be seen from the table, there is an overall improvement of 0.47% for our method compared to the TA method. This indicates that thresholds can be obtained more efficiently using multiple inputs and multiple paths, thus positively affecting model segmentation.

## 5. Conclusions and Future Work

In this paper, we propose the DCOP-Net for few-shot medical image segmentation. To address the issues of prototype bias and low generalization ability in few-shot environments, we design the DFCA and OP modules in the prototype learning stage. These modules enable the support FG features to efficiently learn and integrate new features from the query FG features and enable the extracted prototype to retain crucial contextual information, effectively mitigating the prototype bias problem. In the subsequent segmentation stage, we input the query features into the PTP module to obtain more effective and robust thresholds to delineate the FG and BG regions of the query image. In addition, we propose a query self-reference regularization strategy to optimize the model training process and speed up the model convergence. We validate the effectiveness of DCOP-Net through comprehensive experiments on CHAOS-MRI, Synapse-CT, and CMR datasets. The experimental results show that the proposed model outperforms other state-of-the-art methods on the CHAOS-MRI and Synapse-CT evaluation metrics, demonstrating superior segmentation and generalization capabilities.

Although DCOP-Net achieves strong performance on CHAOS-MRI and Synapse-CT datasets, it does not attain optimal segmentation across all evaluation metrics. For example, it did not outperform the best available method in segmenting LV-MYO on the CMR dataset. This problem is mainly attributed to two factors: (1) the cardiac dataset is difficult to label, resulting in a training dataset with a lower quality than the abdominal dataset, from which the model is difficult to learn high-quality prototypes, and (2) the cardiac dataset has a complex structure, which increases the difficulty of recognition. In addition, the proposed dual-filter cross attention module enhances the utilization of query image foreground features, allowing the model to leverage information from the query image to improve the quality of support prototypes and mitigate prototype bias. However, while the proposed filter cross attention strategy effectively eliminates background mismatches, it also restricts the model’s ability to utilize potentially useful auxiliary information present in the background. Therefore, an important future research direction would be to enhance the utilization of background features by improving intra-class cohesion and inter-class separation in feature space, further optimizing the performance of few-shot segmentation networks. Moreover, in future work, we plan to improve the model’s performance in weakly annotated environments and enhance its ability to handle the segmentation of complex organs for a variety of FSMIS tasks.

## Figures and Tables

**Table 1:** Comparison of different methods under Setting 1 and Setting 2 on CHAOS-MRI and Synapse-CT. Best values are in bold, second best are underlined. ‘–’ indicates not reported.

Setting	Methods	Synapse-CT					CHAOS-MRI				
		Upper		Lower		Mean	Upper		Lower		Mean
		Spleen	Liver	LK	RK		Spleen	Liver	LK	RK	
1	PANet [27]	36.04	49.55	20.67	21.19	32.86	40.58	50.4	30.99	32.19	38.53
	ALP-Net [10]	70.96	78.29	72.36	71.81	73.35	72.18	76.1	71.82	85.18	78.84
	AD-Net [25]	63.48	77.24	72.13	79.06	72.97	72.29	82.11	73.86	85.8	78.51
	AAS-DCL [19]	72.3	78.04	74.58	73.16	74.52	76.24	72.33	80.37	86.11	78.76
	SR&CL [40]	73.41	76.06	73.45	71.22	73.53	76.01	80.23	79.34	87.42	80.77
	CRAPNet [20]	70.37	75.41	74.69	74.18	73.66	74.32	76.46	81.95	86.42	79.79
	Q-Net [26]	–	–	–	–	–	75.99	81.74	78.36	87.98	81.02
	CAT-Net [23]	67.65	75.31	63.36	60.05	66.59	68.83	78.98	74.01	78.9	75.18
	RPT-Net [42]	**79.13**	**82.57**	**77.05**	72.58	**77.83**	76.37	**82.86**	80.72	**89.89**	82.44
	DSP-Net [52]	68.31	69.32	78.01	74.54	72.79	70.93	75.06	81.88	85.37	78.31
	GMRD [14]	56.48	63.06	79.92	62.27	65.43	65.44	73.65	75.97	89.95	65.44
	PAMI [53]	72.38	81.32	76.52	**80.57**	77.69	76.37	82.59	81.83	88.73	82.38
	DCOP-Net	76.06	77.34	72.01	72.97	74.59	**80.52**	78.54	**82.93**	88.58	**82.64**
2	PANet [27]	29.59	38.42	32.34	17.37	29.43	50.9	42.26	53.45	38.64	46.33
	SE-Net [16]	0.23	0.27	32.83	14.34	11.91	51.8	27.43	62.11	61.32	50.66
	ALP-Net [10]	60.25	73.65	63.34	54.82	63.02	67.02	73.05	73.63	78.39	73.02
	AD-Net [25]	50.97	70.63	48.41	40.52	52.63	59.44	77.03	59.64	56.68	63.2
	AAS-DCL [19]	66.36	71.61	64.71	69.95	68.16	74.86	69.94	76.9	83.75	76.36
	SR&CL [40]	67.36	73.63	67.39	63.37	67.94	73.73	75.55	77.07	84.24	77.62
	CRAPNet [20]	70.17	70.45	70.91	67.33	69.72	70.82	73.82	74.66	82.77	75.52
	Q-Net [26]	–	–	–	–	–	65.37	78.25	64.81	65.94	68.59
	RPT-Net [42]	70.8	75.24	**72.99**	**67.73**	71.69	75.46	76.37	78.33	86.01	79.04
	PAMI [53]	71.95	74.13	72.36	67.54	71.49	75.80	**81.09**	74.51	**86.73**	79.53
	DCOP-Net	**73.06**	**77.21**	70.52	66.65	**71.86**	**80.75**	80.35	**80.27**	81.61	**80.74**

**Table 2:** Qualitative comparison of different methods under Setting 1 on the CMR-MRI. The best value is shown in bold font and the second-best value is underlined.

Setting	Methods	CMR				
		LV-BP	LV-MYO	RV	Mean	LV-BP + RV
1	PANet [27]	70.43	46.79	69.52	62.25	139.9
	SE-Net [16]	72.77	44.76	57.13	58.2	129.9
	ALP-Net [10]	83.99	66.74	79.96	76.9	163.95
	AD-net [25]	87.53	62.43	77.31	75.76	164.84
	AAS-DCL [19]	85.21	64.03	79.13	76.12	164.34
	SR&CL [40]	84.74	65.83	78.41	76.32	163.15
	CRAP-net [20]	83.02	65.48	78.27	75.59	161.29
	Q-Net [26]	**90.25**	65.92	78.19	78.15	168.44
	PAMI [53]	89.57	**66.82**	**80.17**	**78.85**	169.74
	DSP-Net [52]	87.75	64.91	79.73	77.46	**169.74**
	DCOP-Net	86.82	60.8	79.48	75.21	166.3

**Table 3:** Ablation experiments with different module combinations on the CHAOS-MRI dataset. Best values are in bold, second best are underlined. ‘–’ indicates not reported.

Methods	Upper		Lower		Mean
	Spleen	Liver	LK	RK	
Baseline	76.97	78.37	73.94	85.93	78.8
Baseline + DFCA	79.25	78.42	77.82	87.97	80.86
Baseline + OP	77.35	78.18	76.03	88.19	79.94
Baseline + QSR	77.52	**79.71**	70.97	87.84	79.01
Baseline + DFCA + OP	79.65	77.55	82.42	88.37	82
Baseline + DFCA + OP + PTP	80.17	78.03	82.47	88.4	82.26
Baseline + DFCA + OP + PTP + QSR	**80.52**	78.54	**82.93**	**88.58**	**82.64**

**Table 4:** Qualitative comparison of the validity of the QSR strategy on the CHAOS-MRI and Synapse-CT datasets. Best values are in bold.

Datasets	Methods	Upper		Lower		Mean
		Spleen	Liver	LK	RK	
CHAOS-MRI	baseline + DFCA + OP + PP	79.86	79.69	80.08	80.19	79.96
	baseline + DFCA + OP + PP + QSR	**80.75**	**80.35**	**80.27**	**81.61**	**80.74**
Synapse-CT	baseline + DFCA + OP + PP	**74.14**	75.06	70.48	65.26	71.24
	baseline + DFCA + OP + PP + QSR	73.06	**77.21**	**70.52**	**66.65**	**71.86**

**Table 5:** Qualitative comparison of the validity of different prior mask generation methods on the CHAOS-MRI dataset. Best values are in bold.

Methods	Upper		Lower		Mean
	Spleen	Liver	LK	RK	
MIFE [23]	**83.44**	**79.49**	79.96	85.89	82.15
PMG	80.52	78.54	**82.93**	**88.58**	**82.64**

**Table 6:** Qualitativecomparison of the validity of different prototype extraction methods on the CHAOS-MRI dataset. Best values are in bold, second best are underlined.

Methods	Upper		Lower		Mean
	Spleen	Liver	LK	RK	
MAP [24]	77.19	78.46	79.92	**88.76**	81.08
ALP [10]	77.8	78.37	80.03	88.39	81.14
RPG [42]	79.29	**78.71**	81.53	87.12	81.66
OP	**80.52**	78.54	**82.93**	88.58	**82.64**

**Table 7:** Ablation study on the maximum number of onion layers. Best values are in bold, second best are underlined.

Nmax	Upper		Lower		Mean
	Spleen	Liver	LK	RK	
1	77.19	78.46	79.92	**88.76**	81.08
3	77.26	**78.71**	81.81	88.3	81.52
4	**80.52**	78.54	**82.93**	88.58	**82.64**
5	80.04	77.56	81.74	88.53	81.97
7	77.88	74.53	81.83	88.56	80.7
9	78.61	72.32	81.67	85.39	78.5

**Table 8:** Qualitative comparison of the validity of different threshold generation modules on the CHAOS-MRI dataset. Best values are in bold.

Methods	Upper		Lower		Mean
	Spleen	Liver	LK	RK	
TA [26]	**83.51**	**79.3**	77.87	87.98	82.17
PTP	80.52	78.54	**82.93**	**88.58**	**82.64**

## Data Availability

Data will be made available on request.
